# Adsorptive Elimination of Heavy Metals from Aqueous Solution Using Magnetic Chitosan/Cellulose-Fe(III) Composite as a Bio-Sorbent

**DOI:** 10.3390/nano13101595

**Published:** 2023-05-10

**Authors:** Aina Mardhia Khalid, Md. Sohrab Hossain, Nor Afifah Khalil, Muzafar Zulkifli, Md. Azharul Arafath, Maizatul Shima Shaharun, Rashid Ayub, Ahmad Naim Ahmad Yahaya, Norli Ismail

**Affiliations:** 1 School of Industrial Technology, Universiti Sains Malaysia (USM), George Town 11800, Malaysia; mardhiakhalid@gmail.com; 2 Department of Fundamental and Applied Sciences, Faculty of Science and Information Technology, Universiti Teknologi PETRONAS, Seri Iskandar 32610, Malaysia; 3 HICoE-Centre for Biofuel and Biochemical Research, Institute of Self-Sustainable Building, Universiti Teknologi PETRONAS, Seri Iskandar 32610, Malaysia; 4 Universiti Kuala Lumpur, Branch Campus Malaysian Institute of Chemical and BioEngineering Technology, Alor Gajah 78000, Malaysia; 5 Department of Chemistry, Shahjalal University of Science and Technology, Sylhet 3114, Bangladesh; 6 Institute of Contaminant Management, Centre for Contaminant Control & Utilization (CenCoU), Department of Fundamental and Applied Sciences, Universiti Teknologi PETRONAS, Seri Iskandar 32610, Malaysia; 7 Department of Chemistry, College of Science, King Saud University, P.O. Box 2455, Riyadh 11451, Saudi Arabia

**Keywords:** adsorption, wastewater treatment, industrial effluent, heavy metals removal, isotherm modeling

## Abstract

Magnetic chitosan/cellulose nanofiber-Fe(III) [M-Ch/CNF-Fe(III)] composites were isolated for the elimination of Cr(VI), Cu(II), and Pb(II) from aqueous solution. Various analytical methods, such as field emission scanning electron microscopy (FE-SEM), transmission electron microscopy (TEM), Fourier-transform infrared spectroscopy (FT-IR), X-ray diffraction analysis (XRD), and thermogravimetric analysis (TGA) were employed to determine the morphological, physicochemical, and thermal properties of the isolated M-Ch/CNF-Fe(III) composites. It was found that the M-Ch/CNF-Fe(III) composites were porous materials, and they have the potential to be implemented as an adsorbent for heavy metals removal. The adsorption efficiency of M-Ch/CNF-Fe(III) composites was determined for Cr(VI), Cu(II), and Pb(II) elimination with changing pH (pH 1.0–8.0), adsorbent doses (0.05–1.0 g), time (15–90 min), and temperature (28–80 °C). In addition, isothermal and kinetics studies were conducted to assess the adsorption behavior and mass transfer phenomena of M-Ch/CNF-Fe(III) composites as an adsorbent for Cr(VI), Cu(II) and Pb(II) elimination from aqueous solution. The outcomes of the present study reveal that the M-Ch/CNF-Fe(III) composites could be utilized as an adsorbent for the Cr(VI), Cu(II), and Pb(II) elimination from industrial effluents.

## 1. Introduction

There is increasing concern regarding the safety of discharging industrial effluents into aquatic ecosystems due to the presence of toxic heavy metals [1]. The presence of heavy metals in an aquatic ecosystem may be detrimental to aquatic life and human health because of their accumulation tendency within living organisms via the food web and their non-degradable nature in the environment [1,2]. Untreated or partially treated industrial effluent discharge is the main reason for heavy metals contamination of aquatic environments [1,3]. Cr(VI), Cu(II), and Pb(II) are the most abundant elements found in aquatic ecosystems because of discharged effluents from various industrial activities, including pigment synthesis, rubber manufacturing, steel manufacturing, the leather industry, tanning, chromite mining, textiles, and the electroplating industry [4,5]. Cr(VI) is considered as a group *A* contaminant and one of the 14 toxic chemicals potentially threatening human health, even at a trace level of contamination [6]. Cu(II) is a destructive toxic element, and its presence in water may cause severe and fatal diseases to aquatic life and humans, including insomnia, Wilson disease, leukemia, and osteoporosis [7,8]. Pb(II) is reported as one of the most toxic heavy metals for human health and aquatic organisms [9]. The presence of Pb(II) in water, even at a low concentration, may cause detrimental effects to human health and to other organisms [9]. Therefore, various environmental protection agencies have suggested allowable concentrations of heavy metals in treated effluents for safe discharge in the aquatic environment. For instance, the Department of Environment (DoE) of Malaysia has set the permitted Cr(VI), Cu(II), and Pb(II) concentrations in treated industrial effluents to 0.05 mg/L, 0.2–1.0 mg/L, and 0.1–0.5 mg/L, respectively [10]. 

Various technologies have been implemented for eliminating heavy metals from industrial effluents. Some of these technologies include electro-flotation [11,12], ion exchange [13], electro-coagulation [14], coagulation–flocculation [15], precipitation [16], membrane filtration [17], and solvent extraction [18]. Although these technologies minimize the residual metal ion concentration in the treated effluent, the high operational cost, materials cost, and low heavy metal uptake efficiency have limited their application in large-scale operations [14,17,19]. Numerous studies have reported that the implementation of a low-cost adsorbent for adsorptive heavy metals elimination from industrial effluents would be an effective alternative to the conventional methods of industrial effluent treatment [8,9,19]. Over the years, various natural bio-polymers, chelating materials, activated carbon, and clay have been utilized as adsorbents for the adsorptive elimination of heavy metals from industrial effluents [8,19,20,21]. For the effective adsorptive elimination of heavy metals, the adsorbent must be biocompatible, economical, and easily separable after adsorption [4,9,19]. The adsorptive elimination of heavy metals from industrial effluents using natural bio-polymers as adsorbents has been viewed as promising technology because of its simplicity in operation, low operating cost, and environmentally friendly nature [4,20,21]. Among the various natural bio-polymers, cellulosic nanofiber (CNF) and chitosan have been widely utilized as adsorbents for the elimination of heavy metal ions from industrial effluents [4,9,19,21]. 

Cellulose is the most abundant natural bio-polymer. CNF obtained from lignocellulosic fiber is a promising bio-sorbent for the adsorptive elimination of heavy metals from industrial effluents because of its renewability, biodegradability, environmentally friendliness, and large surface area, as well as the fact that its carbon chain contains highly reactive primary and secondary hydroxyl groups for binding metal ions [4,22,23]. However, the fragile mechanical strength, poor chemical resistance, poor adsorption efficiency, and low separation efficiency of CNF-based adsorbents after adsorption are the main barrier of utilizing CNF as an adsorbent for the adsorptive elimination of heavy metals from industrial effluents [4]. Chitosan, on the other hand, is the second most abundant natural bio-polymer. Generally, chitosan is a linear polysaccharide, and its carbon chain contains an amino functional group, making chitosan an ideal adsorbent for heavy metals elimination from various effluents [23,24]. However, the poor mechanical strength of chitosan limits its adsorption efficiency of heavy metals. To overcome the prevailing limitations of CNF and chitosan as adsorbents, researchers have suggested the engineered conversion of CNF and chitosan in a magnetic field [4,22,25].

In recent years, magnetic adsorbents have gained extensive interest in the adsorptive elimination of heavy metals from industrial effluents because of their numerous advantages as adsorbents, such as thermo-mechanical strength, chemical resistance, elimination efficiency, regeneration, and environmentally friendly nature [22,25]. Thus, the engineered conversion of chitosan and CNF in a magnetic field would be of considerable interest for effective heavy metals elimination from industrial effluents. In the present study, a chitosan and CNF blend (1:1) was coated with Fe_3_O_4_ to isolate magnetic chitosan/CNF-Fe(III) [M-Ch/CNF-Fe(III)] bio-composite. The M-Ch/CNF-Fe(III) composite was utilized as a bio-sorbent for the adsorption of Pb(II), Cr(VI), and Cu(II) and their elimination from aqueous solution. Various analytical methods were employed to determine the physicochemical, morphological, and thermal properties of the M-Ch/CNF-Fe(III) composite. In addition, the isothermal behavior of the M-Ch/CNF-Fe(III) composite was studied for the adsorptive Cr(VI), Cu(II), and Pb(II) elimination from aqueous solution.

## 2. Materials and Methods

### 2.1. Sample Collection and Preparation

The CNF was isolated from empty oil palm empty fruit bunch fibers following a method discussed elsewhere [4]. FeCl_3_·6H_2_O (purity ≥ 98%) and the ionic liquid 1-butyl-3-methylimidazolim chloride (purity ≥ 99%) were acquired from Sigma Aldrich, St. Louis, MO, USA. K_2_Cr_2_O_7_ (purity ≥ 99%), CuSO_4_·5H_2_O (purity ≥ 98%), and Pb(NO_3_)_2_ were purchased from Merck Chemicals, Selangor, Malaysia. All other chemicals used in the present study were of analytical grade. 

### 2.2. Synthesis M-Ch/CNF-Fe(III) Bio-Composite 

The preparation of the Fe_3_O_4_ powder was conducted using the sol-gel method as discussed elsewhere [4]. Approximately 8 g of sodium acetate and 2 g of FeCl_3_·6H_2_O were mixed with 80 mL of ethylene glycol (C_2_H_6_O_2_). The mixture was then mixed vigorously until the mixture became clear. Subsequently, the mixture was heated for 20 h at 100 °C to obtain a dark brown gel. The obtained gel was washed with C_2_H_5_OH and deionized H_2_O, followed by centrifuging at 5000 rpm for 20 min. The precipitate (Fe_3_O_4_) was collected and dried in a furnace at 300 °C for 15 min. Subsequently, the dried Fe_3_O_4_ was grinned to produce Fe_3_O_4_ powder. Approximately 5 g of chitosan and cellulose blend (1:1) was mixed with 5 mL of 1-butyl-3-methylimidazolium chloride and 2 g of Fe_3_O_4_ powder. The mixture was mixed vigorously for 15 min. Subsequently, the mixture was emulsified by adding Tween 80 (4 mL) and vacuum pump oil (80 mL) at a temperature of 100 °C and a stirring speed of 1000 rpm. The emulsified mixture was then cooled and washed with deionized water and ethanol to remove the residuals. Finally, the synthesized M-Ch/CNF-Fe(III) composite was dried in a vacuum oven and stored at 4 °C prior to characterization and utilization.

### 2.3. Characterization of M-Ch/CNF-Fe(III) Bio-Composite 

The surface morphology of CNF, Fe_3_O_4_, chitosan, and the isolated M-Ch/CNF-Fe(III) bio-composite were determined using SEM (model: Quanta FEG 650, FEI, Hilsboro, OR, USA). In addition, the dimensions and image of CNF isolated from OP-EFB fibers were determined using transmission electron microscopy (TEM) at an accelerating voltage of 80 kV. The functional groups and chemical bonding of CNF, chitosan, and the M-Ch/CNF-Fe(III) composite were determined using Fourier transform infrared (FTIR) spectroscopy within the frequency range of 4000–400/cm. The crystallinity indexes of Fe_3_O_4_, CNF, chitosan, and the M-Ch/CNF-Fe(III) bio-composite were determined by X-ray diffraction (XRD) at 40 kv and 40 mA. The percentage crystallinity indexes (*CrI* %) of Fe_3_O_4_, CNF, chitosan, and the M-Ch/CNF-Fe(III) composite were calculated using Equation (1).

(1) $$CrI(\%)=\frac{I_{200}-I_{am}}{I_{200}}\times 100$$

where *I*_200_ is the intensity of the crystalline and amorphous regions, and *I_am_* is the intensity of the amorphous region. The thermal stabilities of Fe_3_O_4_, chitosan, CNF, and the isolated M-Ch/CNF-Fe(III) composite were determined using thermal gravimetric analysis (TGA). Approximately 10 mg of the sample was heated under a nitrogen atmosphere at a heating rate of 10 °C/min within a heating range from 30 °C to 700 °C. 

### 2.4. Adsorption of Cr(VI), Cu(II), and Pb(II)

The adsorptive Cr(VI), Cu(II), and Pb(II) elimination was conducted using M-Ch/CNF-Fe(III) as an adsorbent with varying adsorbent doses (0.05–1.0 g), times (15–90 min), pH values (pH 1.0–8.0), and temperatures (28–80 °C). The aqueous solution containing Cr(VI), Cu(II), and Pb(II) at concentrations of 100 mg/L, 200 mg/L, and 50 mg/L, respectively, was prepared by dissolving calculated amounts of K_2_Cr_2_O_7_, CuSO_4_·5H_2_O, and Pb(NO_3_) in deionized water, respectively. A certain amount of M-Ch/CNF-Fe(III) and 50 mL of aqueous solution were placed in a 100 mL conical flask, and the mixture was mixed vigorously using a magnetic stirrer. Concentrated NaOH and H_2_SO_4_ solutions were utilized to regulate the pH. The eluent was separated after adsorption using filter paper, and the metal ions’ concentrations in the eluent were determined using atomic adsorption spectroscopy. The percentage adsorptive Cr(VI), Pb(II), and Cu(II) elimination from the aqueous solution using the M-Ch/CNF-Fe(III) composite as an adsorbent was computed as shown in Equation (2).

(2) Removal=Ci-CtCi×100

where *C_i_* and *C_t_* are the Cr(VI), Cu(II), and Pb(II) concentrations (mg/L) initially and at time ‘*t*’, respectively. In addition, the maximum metal ion elimination efficiency (*q_e_*) was computed using Equation (3).

(3) $$q_{e}=\frac{C_{i}-C_{e}}{M}\times V$$

where *M* is the mass of the M-Ch/CNF-Fe(III) composite being used as an adsorbent, *V* is the volume (L) of the aqueous solution, and *C_e_* is the Cr(VI), Pb(II), and Cu(II) concentration (mg/L) at equilibrium. The experiments were conducted in triplicate, and the results are presented in this study as mean values ± standard deviation. 

### 2.5. Adsorption Isotherm 

The Langmuir and Freundlich isotherm models were employed to determine the isothermal behavior for the adsorptive Cr(VI), Cu(II), and Pb(II) elimination from an aqueous solution using the M-Ch/CNF-Fe(III) composite. The adsorption experiments were performed at ambient temperature and pH 5.0 while varying the M-Ch/CNF-Fe(III) composite doses from 0.05 g to 0.5 g as a function of adsorption time from 5 min to 90 min. The best-fitting isotherm model for the adsorptive Cr(VI), Cu(II), and Pb(II) elimination using the M-Ch/CNF-Fe(III) composite was predicted based on the regression coefficient (*R*^2^) values of Langmuir and Freundlich isotherm models. The linear forms of the Langmuir and Freundlich isotherm model equations can be written as shown in Equation (4) and Equation (5), respectively [26].

(4) $$\frac{1}{q_{e}}=\frac{1}{abC_{e}}+\frac{1}{b}\;$$

(5) $$\log q_{e}=\log K_{f}+\frac{1}{n}\log C_{e}$$

where *a* refers to the Langmuir constant, *b* expresses the maximum adsorption value, *K_f_* (L/mg) denotes to the Freundlich affinity constant, and *n* is the Freundlich exponential constant. 

### 2.6. Adsorption Kinetics 

The kinetic behavior for the Cu(II), Cr(VI), and Pb(II) elimination from aqueous solution was determined using pseudo-1st-order and pseudo-2nd-order kinetics model equations. The experiments were performed at varying temperatures from ambient temperature (28 ± 1 °C) to 70 °C as a function of time from 5 min to 60 min at pH 5.0 and adsorbent doses of 0.25 mg. The suitability of the best-fitting kinetics model equation was assessed by comparing the *q_e_* and *R*^2^ values. The linear forms of the pseudo-1st- and 2nd-order kinetics equations are presented in Equation (6) and Equation (7), respectively [26].

(6) $$\ln\left(q_{e}-q_{t}\right)=\ln q_{e}-k_{1}t\;$$

(7) $$\frac{t}{q_{e}}=\frac{1}{k_{2}q_{e}^{2}}+\frac{t}{q_{e}}\;$$

where *q_e_* and *q_t_* represent the adsorption efficiency at equilibrium and at adsorption time ‘*t*’, respectively. In addition, *k*_1_ and *k*_2_ denote the adsorption rate constant of the psuedo-1st-order and pseudo-2nd-order kinetics model equations for Cr(VI), Cu(II), and Pb(II) elimination using the M-Ch/CNF-Fe(III) composite as an adsorbent. 

### 2.7. Desorption Studies

The reusability of the M-Ch/CNF-Fe(III) composite was determined via adsorption/desorption of Cr(VI) from aqueous solution for 6 cycles. The adsorption experiments were conducted for the elimination of Cr(VI) using the M-Ch/CNF-Fe(III) composite at a Cr(VI) concentration of 100 mg/L and adsorbent doses of 0.5 g/L, with the pH being 4.0 and at ambient temperature for an adsorption time of 30 min. After adsorption, the adsorbent was separated using a magnet, and the separated adsorbent was placed in 50 mL of 0.5 M HCl solution for the desorption of the adsorbed Cr(VI). The mixture was then vigorously stirred using a magnetic stirrer for 30 min. Subsequently, the adsorbent was separated and washed with deionized water prior to reuse for the subsequent adsorption/desorption experiments. 

## 3. Results

### 3.1. Characterization of M-Ch/CNF-Fe(III) Composite

**Figure 1:**
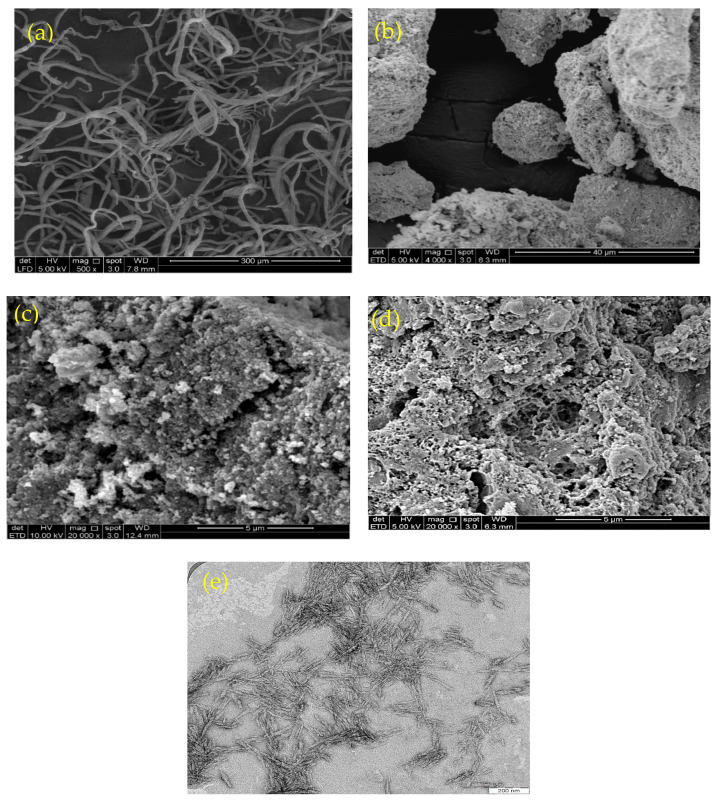
Scanning electron microscope image of CNF (**a**), Fe_3_O_4_ (**b**), chitosan (**c**), and M-Ch/CNF-Fe(III) composite (**d**), and TEM image of CNF (**e**).

**Figure 2:**
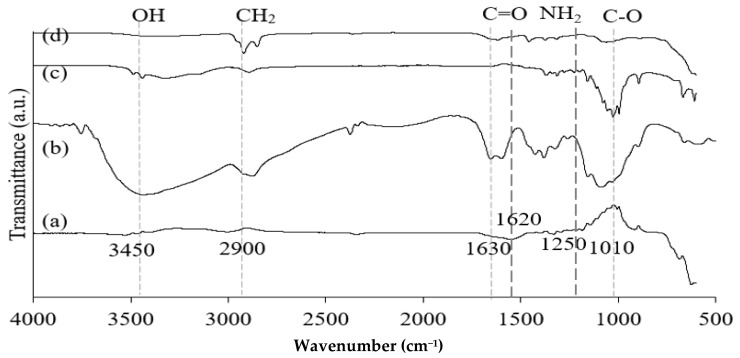
FT-IR spectra of Fe_3_O_4_ (**a**), chitosan (**b**), CNF (**c**), and M-Ch/CNF-Fe(III) composite (**d**).

Figure 1 shows scanning electron microscopy (SEM) images of CNF (Figure 1a), magnetite (Figure 1b), chitosan (Figure 1c), and the isolated M-Ch/CNF-Fe(III) composite (Figure 1d), as well as a TEM image of CNF (Figure 1e). Figure 1a shows that the surface morphology of the CNF was smooth, with little agglomeration. The obtained smooth surface of CNF reveals that the soda pulping, bleaching, and acid hydrolysis process effectively removed the lignin, hemicellulose, and extractives from OP-EFB fibers. The fiber agglomeration obtained might be due to the surface ionic charge between H^+^ and *SO*_4_^2−^ during the acid hydrolysis process using sulfuric acid [27,28]. The diameter of the isolated CNF was determined to be 8–15 nm. Similarly, Fatah et al. [28] reported a diameter of 5–10 nm of CNF isolated from OP-EFB using chemo-mechanical methods. The SEM image of Fe3O_4_ shows a rough surface with a diameter of 100–200 nm. However, the SEM image of the M-Ch/CNF-Fe(III) composite shows the surface morphology of the isolated M-Ch/CNF-Fe(III) composite was porous and rough, with irregular shapes (Figure 2d). The rough surface of the isolated M-Ch/CNF-Fe(III) composite was obtained because of the aggregation among the particles of CNF, chitosan, and Fe3O_4_ [9,29]. Studies have reported that porous materials with a high surface area are promising adsorbents for the adsorptive elimination of heavy metals [30]. Therefore, the isolated porous M-Ch/CNF-Fe(III) composite has the desired properties to be utilized as an adsorbent for adsorptive heavy metals removal, including of Cr(VI), Cu(II), and Pb(II). 

Figure 2 shows the FT-IR spectra of Fe_3_O_4_ particles, chitosan, CNF, and the isolated M-Ch/CNF-Fe(III) composite. The broad absorption peaks obtained for chitosan and CNF approximately at 3420/cm correspond to N-H and O-H bonds, respectively [31]. The absorption peak at 2900/cm is attributed to C-H stretching. The adsorption peaks on the chitosan and M-Ch/CNF-Fe(III) composite spectra at 1630/cm are attributed to the bending vibrations of H-O-H for adsorbed water [32,33]. However, the characteristic peak at 1500/cm is assigned to the C=O bonds present on the surface of chitosan and the M-Ch/CNF-Fe(III) composite [33]. Ge and Hua [23] obtained FTIR spectra for poly(maleic acid)-grafted crosslinked chitosan nanoparticles and observed that the major bands were an O-H stretching band at 3506/cm, an N-H stretching band at 3294/cm, a C-H stretching band at 2874/cm, and a C-O stretching band at 1048/cm. However, the absence of adsorption peaks at 1740/cm and 460/cm on the CNF spectra reveals that the CNF isolation processes, such as pulping, bleaching, and acid hydrolysis, effectively removed hemicellulose and lignin from the OP-EFB fiber [4]. However, the presence of a tiny adsorption band on the Fe_3_O_4_ spectrum at 3420/cm reveals the existence of O-H bond. The presence of an O-H bond on the Fe_3_O_4_ spectrum was due to the presence of ethylene glycol, which was utilized in the formation of Fe_3_O_4_ from FeCl_3_·4H_2_O. The existence of the adsorption peak at 595/cm on the spectra of Fe_3_O_4_ and the M-Ch/CNF-Fe(III) composite reveals the formation of Fe-O bonds on the surface of the M-Ch/CNF-Fe(III) composite [24]. Similarly, Khalid et al. [4] identified an O-H stretching vibration band at 3420/cm on the surface of an OP-EFB nanofiber and a magnetic OP-EFB nanofiber composite. 

**Figure 3:**
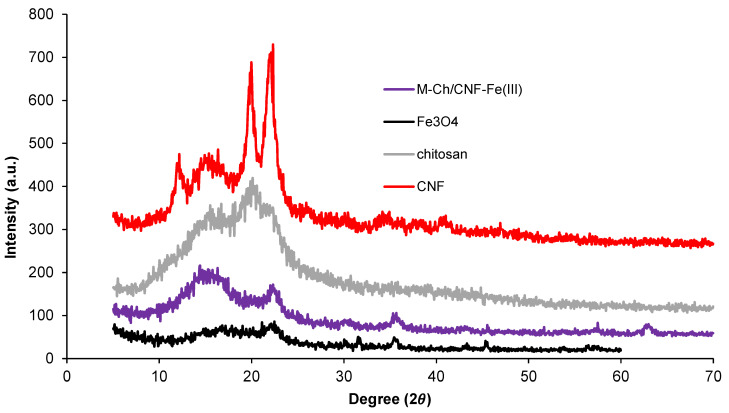
Crystallinity index using XRD analyses of Fe_3_O_4_, chitosan, CNF, and M-Ch/CNF-Fe(III) composite.

X-ray diffraction (XRD) patterns of Fe_3_O_4_, chitosan, CNF, and the M-Ch/CNF-Fe(III) composite are presented in Figure 3. Two peak intensities were observed at 16° and 22° on the XRD patterns of CNF, which indicates that the isolated CNF was of the cellulose I type [28]. The crystalline index (%) of CNF was calculated to be 69%. Generally, the XRD of chitosan reveals peaks at 9–10° and 19–20°. In the present study, the chitosan peak was found at 19.5°, which is similar to studies reported by Karimi [22] and Rahimi et al. [25]. The XRD pattern of Fe_3_O_4_, which is isolated from FeCl_3_·4H_2_O, shows peaks at 30.1°, 35.5°, 47.4°, and 53.2°. Similar peaks observed in the isolated M-Ch/CNF-Fe(III) composite, revealing that the Fe_3_O_4_ was successfully introduced to the isolated M-Ch/CNF-Fe(III) composite. Similar observations were reported by Rahimi et al. [25] and Ge and Hua [23]. 

**Figure 4:**
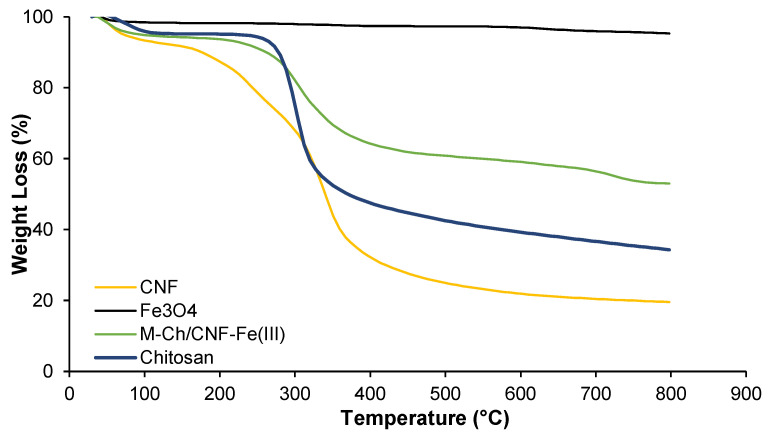
TGA analyses of Fe_3_O_4_, chitosan, CNF, and M-Ch/CNF-Fe(III) composite.

Figure 4 shows the TGA analyses of chitosan, Fe_3_O_4_, CNF, and the M-Ch/CNF-Fe(III) composite. It was observed that the thermal decomposition of the M-Ch/CNF-Fe(III) composite, CNF, and chitosan occurred at two stages. The first stage of decomposing occurred at a temperature from 28–173 °C, 28–257 °C, and 28–239 °C for the CNF, chitosan, and M-Ch/CNF-Fe(III) composite, respectively. The second stage of decomposition occurred at a temperature from 173–408 °C, 257–362 °C, and 239–328 °C for CNF, chitosan, and the M-Ch/CNF-Fe(III) composite, respectively. The onset temperature (T_onset_) and the maximum degradation temperature (T_max_) for chitosan, Fe_3_O_4_, CNF, and the M-Ch/CNF-Fe(III) composite are shown in Table 1. It was found that the T_onset_ of the M-Ch/CNF-Fe(III) composite was lower than that of chitosan, but it was higher than that of CNF, revealing the successful incorporation of Fe_3_O_4_ particles into the M-Ch/CNF-Fe(III) composite [9]. However, the T_max_ for the M-Ch/CNF-Fe(III) composite was lower than those of chitosan and CNF. The weak intermolecular and intramolecular hydrogen bonding between the matrices in the M-Ch/CNF-Fe(III) composite might lessen the maximum degradation temperature. Similarly, Zhu et al. [34] further reported that the lesser T_max_ of the M-Ch/CNF-Fe(III) composite does not influence the adsorptive elimination of metal ions, as most of the adsorptive elimination of heavy metals and other contaminants is conducted at ambient temperature. The percentage weight loss of the M-Ch/CNF-Fe(III) composite was determined to be 50%, which is lower than the weight loss values of chitosan and cellulose, indicating that Fe_3_O_4_ was successfully incorporated with CNF and chitosan during the formation of the M-Ch/CNF-Fe(III) composite.

### 3.2. Adsorption of Cr(VI), Cu(II), and Pb(II) Using M-Ch/CNF-Fe(III) Composite

**Figure 5:**
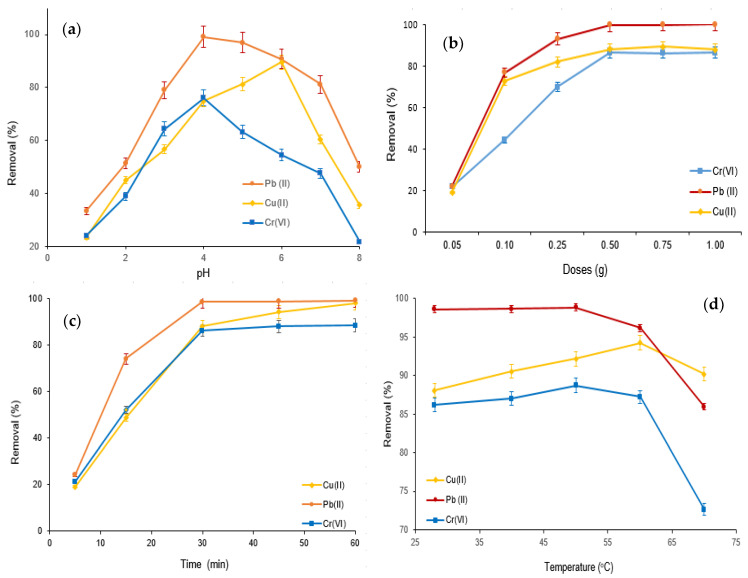
Adsorptive elimination of Cr(VI), Cu(II), and Pb(II) from aqueous solution using M-Ch/CNF-Fe(III) composite as an adsorbent. (**a**) Effect of pH, (**b**) effect of adsorbent doses, (**c**) effect of adsorption time, and (**d**) effect of temperature.

The adsorptive elimination of Cr(VI), Cu(II), and Pb(II) using the M-Ch/CNF-Fe(III) composite as an adsorbent was carried out with varying pH values, adsorbent doses, adsorption times, and temperatures, as shown in Figure 5. The influence of pH on the Cr(VI), Cu(II), and Pb(II) elimination was examined with varying pH values (pH 1.0 to pH 8.0) at an adsorption dose of 0.25 g, with an adsorption time of 30 min and at ambient temperature (28 ± 1 °C). It was observed that the percentage of adsorptive Cr(VI), Cu(II), and Pb(II) elimination increased with increasing pH from pH 1.0 to pH 4.0 for Cr(VI) and Pb(II) removal, and pH 1.0 to pH 6.0 for Cu(II) elimination (Figure 5a). However, the adsorptive Cr(VI) and Pb(II) elimination decreased with increasing pH over pH 4.0, and the percentage elimination of Cu(II) decreased with increasing pH over pH 5.0. The highest Cu(II) elimination obtained was about 86% at pH 6.0, with an adsorption time of 30 min and adsorption doses of 0.25 g at ambient temperature. The Cr(VI) and Pb(II) elimination values obtained were about 76% and 99%, respectively, at pH 4.0 and ambient temperature with an adsorption time of 30 min and adsorption doses of 0.25 g. Generally, pH is the most influential variable in the adsorptive elimination of contaminants from wastewater [13,35]. The reactive functional groups on the surface of the adsorbent modify with the alteration of pH, which substantially influences the adsorptive elimination of contaminants [36]. The more abundant H^+^ in aqueous solution at a lower pH competes with metal ions to bind to the surface of the M-Ch/CNF-Fe(III) composite, resulting in minimized Cu(II), Pb(II), and Cr(VI) removal. However, the amount of H^+^ decreases with increasing pH, which substantially increases deprotonation of the reactive functional groups on the surface of the M-Ch/CNF-Fe(III) composite and therefore increases the Cu(II), Pb(II), and Cr(VI) removal. However, the decreases of Pb(II) and Cr(VI) elimination over pH 4.0 and Cu(II) elimination over 6.0 can be accredited to the electrostatic repulsion between the reactive functional groups on the surface of the M-Ch/CNF-Fe(III) composite and metal ions due to the increased protonation at higher pH. The present study’s findings agree with the studies reported by Dong et al. [37] and Mohamed et al. [35].

The influence of adsorbent doses on the Cu(II), Cr(VI), and Pb(II) elimination using the M-Ch/CNF-Fe(III) composite as an adsorbent was determined by varying the adsorbent doses from 0.05 g to 1.0 g at pH 4.0 and ambient temperature, using an adsorption time of 30 min, as shown in Figure 5b. The results show that the percentages of Cu(II), Cr(VI), and Pb(II) elimination improved with increasing M-Ch/CNF-Fe(III) composite doses from 0.05 g to 0.5 g, and declined with increases of the M-Ch/CNF-Fe(III) composite doses over 0.5 g. The maximum values of about 86%, 100%, and 88% for Cr(VI), Pb(II), and Cu(II) removal, respectively, were obtained at adsorbent doses of 0.5 g, with an adsorption time of 30 min and at pH 4.0. The escalation of the Cu(II), Cr(VI), and Pb(II) elimination with increasing M-Ch/CNF-Fe(III) composite doses can be referred to as the increase of the reactive group for binding metal ions [35]. The decrease of Cu(II), Cr(VI), and Pb(II) elimination with M-Ch/CNF-Fe(III) composite doses over 0.5 g was due to the saturation of the Cu(II), Cr(VI), and Pb(II) concentrations with reactive groups on the surface of the M-Ch/CNF-Fe(III) composite [35,38]. In addition, the particle aggregation of M-the Ch/CNF-Fe(III) composite with higher amounts of adsorbent doses over 0.5 g could be another reason for the declining increase of Cu(II), Cr(VI), and Pb(II) adsorption [38]. 

Adsorptive Cr(VI), Cu(II), and Pb(II) elimination using the M-Ch/CNF-Fe(III) composite as an adsorbent was assessed with varying treatment times from 5–90 min at pH 4.0, using adsorbent doses of 0.5 g at room temperature, as presented in Figure 5c. The percentages of Cr(VI), Cu(II), and Pb(II) elimination increased with increasing adsorption time and reached their maximum values at 30 min; thereafter, the percentages of Cr(VI), Cu(II), and Pb(II) elimination were found to be negligible with the further increase of adsorption time over 30 min. The highest values of about 86%, 99%, and 88% for Cr(VI), Pb(II), and Cu(II) elimination were obtained at an adsorption time of 30 min at pH 4.0, with adsorbent doses of 0.5 g at ambient temperature. The negligible increase of Cr(VI), Pb(II), and Cu(II) elimination using the M-Ch/CNF-Fe(III) composite as an adsorbent over 30 min of adsorption time could be attributed to the saturation of active functional groups on the surface of the adsorbent with metal ions. The observations were found to be similar, to those reported by Vishnu et al. [39] and Mat Yasin et al. [40]. Vishnu et al. [39] obtained the maximum Cu(II), Cr(VI), and Pb(II) elimination at an adsorption time of 45 min using magnetic microspheres of *Muraya koenigii* extract.

The effects of temperature on Cr(VI), Cu(II), and Pb(II) elimination using the M-Ch/CNF-Fe(III) composite as an adsorbent were evaluated by varying the temperature from ambient temperature to 80 °C at pH 4.0, with an adsorption time of 30 min and adsorbent doses of 0.5 g, as shown in Figure 5d. As can be seen in Figure 5d, the Cr(VI) adsorption was slightly increased with increasing temperature up to 50 °C, and thereafter the Cr(VI) adsorption was found to decrease with increasing temperature. Similar adsorption behavior was found for Cu(II) removal. Cu(II) adsorption increased with increasing the temperature from ambient temperature to 60 °C and decreased after that. In the case of Pb(II) adsorption, the increase in temperature did not influence the Pb(II) elimination up to 50 °C. However, the Pb(II) adsorption was decreased with increasing temperature over 50 °C. The increases of Cr(VI) and Cu(II) elimination with increasing temperature was occurred might be due to the increasing reactive groups on the surface of the M-Ch/CNF-Fe(III) composite with increasing temperature. However, the decreases of Cr(VI) and Pb(II) elimination was found with elevated temperature over 50 °C were due to the weakened chemical bonding within the matrices of the M-Ch/CNF-Fe(III) composite, which lessened the affinity of binding metal ions and therefore decreased the Cr(VI) and Pb(II) removal. Similarly, Fan et al. [41] observed that the adsorptive elimination of Pb(II), Zn(II), and Cd(II) increased with increasing temperature because of increasing active functional groups on the surface of *Penicillium simplicissimum*. 

### 3.3. Adsorption Equilibrium Isotherm for Cr(VI), Cu(II), and Pb(II) Elimination 

The evaluation of the adsorption equilibrium is essential to assess the adsorption behavior for adsorptive heavy metals removal. Over the years, various isotherm model equations have been used to determine the adsorption behavior for the elimination of heavy metals [37,41], dyes [27], and other organic and inorganic contaminants [40] from various types of wastewater. The Langmuir and Freundlich isotherm models were implemented in the present study to determine the adsorption behavior for Cr(VI), Cu(II), and Pb(II) elimination using the M-Ch/CNF-Fe(III) composite as an adsorbent. The Langmuir isotherm model expresses that the adsorptive contaminant elimination occurs on the surface of the adsorbent in a monolayer formation and with a uniform distribution [35]. Conversely, the Freundlich isotherm model states that the adsorptive elimination ensues on the heterogenous surface of the adsorbent in a multilayer formation and with a non-uniform distribution. However, the Freundlich isotherm model does not limit the uniform distribution of the contaminant on the surface of the adsorbent during the adsorptive contaminant elimination [41]. 

**Figure 6:**
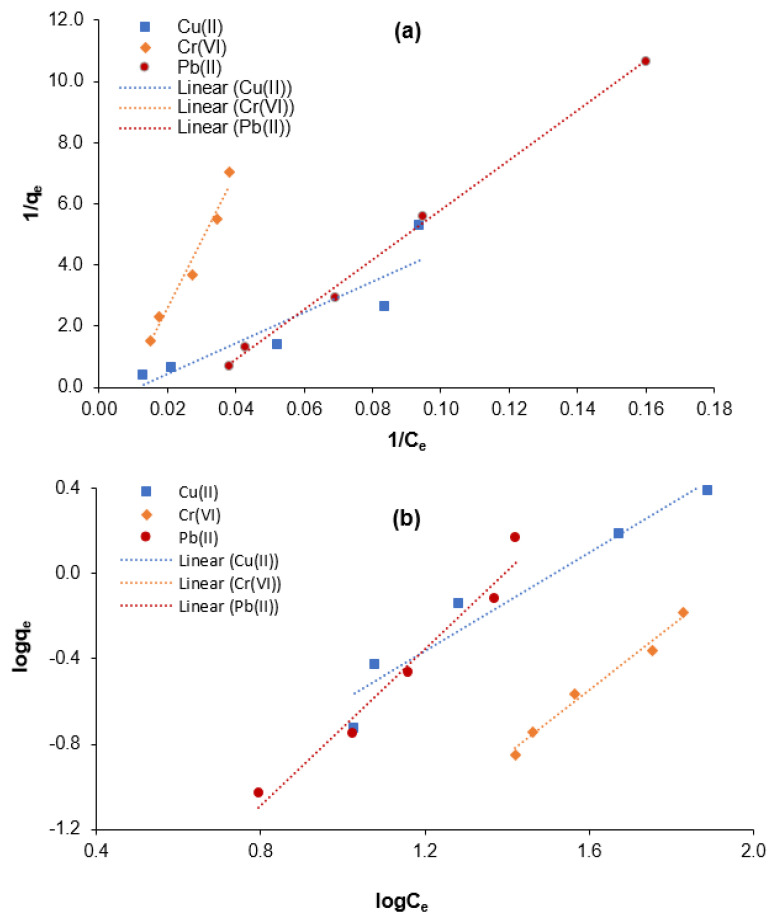
Isotherm modeling for the adsorptive elimination of Cr(VI), Cu(II), and Pb(II) using M-Ch/CNF-Fe(III) composite as an adsorbent. (**a**) Langmuir isotherm model and (**b**) Freundlich isotherm model.

The isothermal behavior for adsorptive Cr(VI), Cu(II), and Pb(II) elimination from aqueous solution using the M-Ch/CNF-Fe(III) composite as an adsorbent was determined as shown in Figure 6. The Freundlich exponential constant (*n*), Langmuir constant (*a*), Freundlich affinity constant (*K_f_*), maximum adsorption (*b*), and correlation coefficient (*R*^2^) values were calculated for Cr(VI), Cu(II), and Pb(II) elimination from aqueous solution using the M-Ch/CNF-Fe(III) composite as an adsorbent and are shown in Table 2. It was found that the positive Langmuir constant (*a*) and maximum adsorption (*b*) values reveal the applicability of the M-Ch/CNF-Fe(III) composite as an adsorbent for the elimination of Cr(VI), Cu(II), and Pb(II) from aqueous solution. The maximum adsorption values were determined to be 1.7655 mg/mg, 0.5412 mg/mg, and 0.4232 mg/mg for the elimination of Cu(II), Cr(VI), and Pb(II), respectively. 

The correlation coefficient (*R*^2^) values were utilized to determine the suitable isotherm models for assessing the adsorption behavior of the M-Ch/CNF-Fe(III) composite for Cr(VI), Cu(II), and Pb(II) removal. It was found that the *R*^2^ values for the elimination of Cu(II) and Cr(VI) were 0.8228 and 0.9677 for the Langmuir isotherm model, respectively, and 0.9378 and 0.9825 for the Freundlich isotherm model, respectively. The higher *R*^2^ values for the Freundlich isotherm model reveal that the Freundlich isotherm model is the best-fitting isotherm model for the elimination of Cu(II) and Cr(VI) using the M-Ch/CNF-Fe(III) composite as an adsorbent. The *R*^2^ values for Pb(II) elimination were determined to be 0.9967 and 0.9668 for the Langmuir and Freundlich isotherm models, respectively. Based on the *R*^2^ values, it can be postulated that both the Langmuir and Freundlich isotherm models can describe the adsorption behavior of Pb(II) using the M-Ch/CNF-Fe(III) composite because the *R*^2^ value is ˃0.96 for both isotherm models. However, the Langmuir isotherm model would be the best-fitting isotherm model for the adsorptive Pb(II) elimination because of the higher *R*^2^ value. The findings are in agreement with those reported by Razak et al. [8] and Lian et al. [9]. Razak et al. [8] reported the Freundlich isotherm model was the best-fitting isotherm model for Cu(II) elimination from industrial wastewater effluent using chemically modified kenaf fiber as an adsorbent. Lian et al. [9] observed that the Langmuir isotherm model was the most suitable model for describing the adsorption behavior of Pb(II) using EDTA-functionalized magnetic chitosan oligosaccharides as an adsorbent. 

### 3.4. Adsorption Kinetics for Cr(VI), Cu(II), and Pb(II) Elimination 

The mass transfer phenomena and adsorption mechanisms of the M-Ch/CNF-Fe(III) composite as an adsorbent for the elimination of Pb(II), Cu(II), and Cr(VI) were determined using pseudo-first-order and pseudo-second-order kinetics model equations, as depicted in Table 3. It was noticed that the experimental adsorption efficiency (*q_e_* (exp)) values increased with increasing adsorption temperature from room temperature (28 ± 1 °C) to 70 °C, revealing that the exterior energy influenced the adsorption efficiency of the M-Ch/CNF-Fe(III) composite for Pb(II), Cu(II), and Cr(VI) elimination from aqueous solution [26,42]. The comparison of the *R*^2^ values and the difference between the *q_e_* (exp) and *q_e_* (theoretical) values obtained from the pseudo-first-order and pseudo-second-order kinetics model equations showed that the pseudo-second-order model was the best-fitting kinetics equation to describe the mass transfer phenomena for the elimination of Pb(II), Cr(VI), and Cu(II) using the M-Ch/CNF-Fe(III) composite as an adsorbent. Thus it can be postulated that chemisorption is a possible mechanism for the elimination of Cu(II), Pb(II), and Cr(VI) from an aqueous solution using the M-Ch/CNF-Fe(III) composite as an adsorbent. Similarly, Sun et al. [42] and Liu et al. [43] found that the pseudo-second-order kinetic equation was the best-fitting kinetics model for Cr(VI) elimination using an amino-functionalized magnetic CNF composite and magnetic S-doped Fe-Cu-La trimetallic oxide as magnetic adsorbents, respectively. 

### 3.5. Reusability of M-Ch/CNF-Fe(III) Composite 

**Figure 7:**
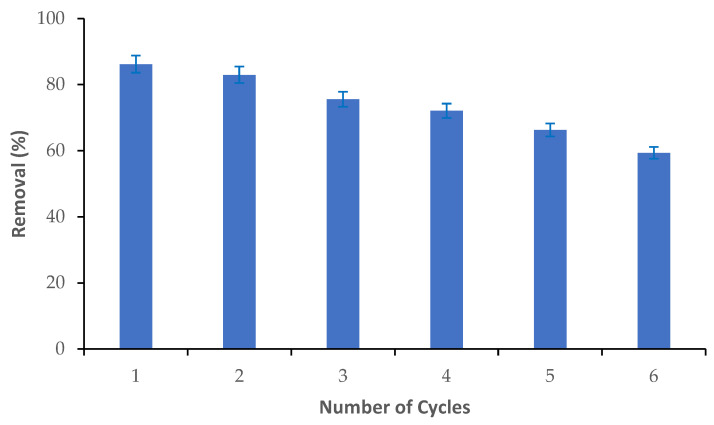
Reusability of M-Ch/CNF-Fe(III) composite for the elimination of Cr(VI) from aqueous solution.

The reusability of the M-Ch/CNF-Fe(III) composite was determined via the adsorption and desorption of Cr(VI) from an aqueous solution, as shown in Figure 7. It was observed that the Cr(VI) adsorption efficiency slightly reduced from cycle 1 to cycle 2, and sharply reduced thereafter. Approximately 86% of the Cr(VI) elimination was obtained during cycle 1. However, the percentage of Cr(VI) adsorption was reduced to about 83% in cycle 2, and it was substantially reduced to about 59% in cycle 6. The decrease of the Cr(VI) adsorption efficiency with an increasing number of adsorption/desorption cycles could be attributed to the breakdown of the structure of the M-Ch/CNF-Fe(III) composite and the loss of active functional groups on the surface of the M-Ch/CNF-Fe(III) composite [43,44]. Thus, the fabricated M-Ch/CNF-Fe(III) composite has the potential to be recycled and reused for the elimination of metal ions from aqueous solution. Similarly, Daneshfozoun et al. [44] observed that the magnetic cellulosic nanofiber can be recycled and reused for the elimination of heavy metal ions. Anush and Vishalakshi [45] reported that magnetic nanoparticles incorporated with chitosan gel had the potential to be recovered and reused for the elimination of Cr(VI) and Cr(II) from aqueous solution. 

Table 4 shows the metal ion uptake efficiency of various magnetic adsorbents from aqueous solution. Based on the findings of the present study, it can be postulated that the isolated M-Ch/CNF-Fe(III) composite has the potential to be utilized as an absorbent for the elimination of heavy metal ions from aqueous solution, including Cu(II), Cr(VI), and Pb(II). The highest metal ion uptake efficiency values of the M-Ch/CNF-Fe(III) composite obtained were 391.21 mg/g, 185.21 mg/g, and 99.86 mg/g for the elimination of Cu(II), Cr(VI), and Pb(II), respectively. Zhang et al. [46] obtained a Cr(VI) uptake efficiency of about 280 mg/g using a polyethyleneimine-functionalized Fe_3_O_4_/steam-exploded rice straw composite as an adsorbent. In addition, Touihri et al. [47] obtained maximum Cr(VI) and Cu(II) elimination efficiencies of 212.22 mg/g and 68.64 mg/g, respectively, using magnetic pinecone gel beads as an adsorbent. However, the fabricated M-Ch/CNF-Fe(III) composite showed better adsorption efficiency of heavy metal ions than silica-coated amino-functionalized magnetic *Muraya koenigii* extracts [39], magnetic nanoparticles incorporated with chitosan gel [45], magnetically activated carbon nanoparticles [48], and magnetically modified alkali-conditioned anaerobically digested sludge [49]. 

## 4. Conclusions

The present study isolated an M-Ch/CNF-Fe(III) composite as an effective adsorbent for Pb(II), Cu(II), and Cr(VI) elimination from aqueous solution. Surface morphology analyses reveal that the isolated M-Ch/CNF-Fe(III) composite is porous and rough, with irregular shapes. FT-IR, XRD, and TEM analyses showed that chitosan, CNF, and Fe_3_O_4_ were successfully incorporated during the formation of the M-Ch/CNF-Fe(III) composite. The pH, adsorbent doses, and adsorption time potentially influenced the adsorptive Pb(II), Cu(II), and Cr(VI) elimination using the M-Ch/CNF-Fe(III) composite as an adsorbent. Maximum values of about 86%, 100%, and 88% for Cr(VI), Pb(II), and Cu(II) elimination, respectively, were obtained at adsorbent doses of 0.5 g, pH 4.0, an adsorption time of 30 min, and ambient temperature. Isothermal studies showed that the Freundlich isotherm model was the best-fitting isotherm model for adsorptive Cu(II) and Cr(VI) elimination. The Langmuir isotherm model was the best-fitting isotherm model for removing Pb(II) from aqueous solution. Kinetics studies showed that chemisorption was the mass transfer mechanism of the M-Ch/CNF-Fe(III) composite as an adsorbent for Pb(II), Cu(II), and Cr(VI) elimination from aqueous solution. Based on the present study’s findings, it can be postulated that the isolated M-Ch/CNF-Fe(III) composite can be implemented to remove heavy metals and other contaminants from industrial effluents. 

## Figures and Tables

**Table 1:** Thermal properties of Fe_3_O_4_, chitosan, CNF, and M-Ch/CNF-Fe(III) composite.

Materials	T_onset_ (°C)	T_max_ (°C)	Weight Loss (%)
Fe_3_O_4_	700	750	5
Chitosan	257	362	66
CNF	173	408	81
M-Ch/CNF-Fe(III)	239	328	50

**Table 2:** Adsorption isotherm modeling for the elimination of Cu(II), Cr(VI), and Pb(II) from aqueous solution using M-Chi-CNF composite as an adsorbent.

	Langmuir Isotherm			Freundlich Isotherm		
	*R* ^2^	*a* (L/mg)	*b* (mg/mg)	*R* ^2^	*k_f_* (L/mg)	*n*
Cu(II)	0.8228	0.0113	1.7655	0.9378	0.0179	0.8670
Cr(VI)	0.9677	0.0084	0.5412	0.9825	0.0011	0.6628
Pb(II)	0.9967	0.0001	0.4232	0.9668	0.0028	0.5453

**Table 3:** Kinetics modeling for Cr(VI), Cu(II), and Pb(II) elimination from aqueous solution using M-Ch/CNF-Fe(III) composite as an adsorbent.

Metal Ions	Temperature (°C)		Pseudo-1st-Order			Pseudo-2nd-Order		
		*q_e_* (exp) (mg/mg)	*q_e_* (mg/mg)	*K*_1_ (1/min)	*R* ^2^	*q_e_* (mg/mg)	*K*_2_ (mg/mg.min)	*R* ^2^
Cu(II)	28	2.669	3.782	0.056	0.9482	2.869	0.073	0.9985
	40	2.715	3.749	0.061	0.9896	2.914	0.098	0.9999
	50	2.734	3.685	0.072	0.9971	2.989	0.198	0.9998
	60	2.784	3.402	0.079	0.9982	3.029	0.207	0.9999
	70	2.815	3.309	0.076	0.9865	3.196	0.218	0.9999
Cr(VI)	28	4.496	5.435	0.024	0.9726	4.997	0.092	0.9996
	40	4.498	5.102	0.014	0.9896	5.089	0.106	0.9901
	50	5.084	4.253	0.042	0.9069	5.172	0.133	0.9864
	60	5.106	4.256	0.048	0.9648	5.281	0.147	0.9984
	70	5.109	3.831	0.049	0.9907	5.328	0.149	0.9995
Pb(II)	28	5.590	6.424	0.042	0.9242	5.475	0.016	0.9996
	40	5.596	5.557	0.043	0.9891	5.573	0.075	0.9979
	50	5.604	5.342	0.058	0.9556	5.585	0.132	0.9993
	60	5.619	5.046	0.068	0.9979	5.593	0.295	0.9998
	70	5.627	4.443	0.096	0.9663	5.594	0.306	0.9999

**Table 4:** Reported studies for the elimination of heavy metals ion from aqueous solution using magnetic adsorbent.

Magnetic Adsorbent	Metals Ion	Removal Capacity (mg/g)	References
Silica-coated amino-functionalized magnetic *Muraya koenigii* extracts	Cr(VI) Cu(II)	71.12 73.71	[39]
Magnetic nanoparticles incorporated with chitosan gel	Cr(VI) Cu(II)	83.33 90.90	[45]
Magnetic pinecone gel beads	Cr(VI) Cu(II)	212.22 68.64	[47]
Magnetically activated carbon nanoparticles	Cr(VI) Cu(II) Pb(II)	2.38 0.74 0.25	[48]
Polyethylenimine-functionalized Fe_3_O_4_/steam-exploded rice straw composite	Cr(VI)	280.11	[46]
Magnetically modified alkali-conditioned anaerobically digested sludge	Cu(II) Cd(II) Pb(II)	29.72 28.55 28.60	[49]
Modified magnetic metal–organic framework	Hg(II) Cd(II) Pb(II)	431 393 397	[50]
Functionalized double-modified covalent organic framework	Pb(II)	14.22	[51]
M-Ch/CNF-Fe(III) composite	Cu(II) Cr(VI) Pb(II)	391.21 185.22 99.86	Present study

## Data Availability

Not applicable.
